# Perspectives of pregnant women on the utilisation of a maternity waiting home near Onandjokwe Lutheran Hospital in Oshikoto Region, Namibia

**DOI:** 10.4102/safp.v66i1.5943

**Published:** 2024-05-14

**Authors:** Daniel O. Ashipala, Medusalem H. Joel, Louise Pretorius

**Affiliations:** 1 Department of General Nursing Sciences, Faculty of Health Sciences and Veterinary Medicine, University of Namibia, Rundu, Namibia; 2 Department of General Nursing Sciences, Faculty of Health Sciences and Veterinary Medicine, University of Namibia, Windhoek, Namibia

**Keywords:** enablers, barriers, maternity waiting home, utilisation, pregnant women, maternal and child health

## Abstract

**Background:**

Despite the efforts of Namibia’s Ministry of Health and Social Services to build maternity waiting homes (MWHs), few pregnant women make use of them. Long distances among the general population in Namibia limit the utilisation of MWHs. Little research has investigated what factors are limiting the use of these facilities despite the urgent need for them. The aim of this study thus was to explore and describe the perspectives of pregnant women on the utilisation of the MWHs near Onandjokwe Lutheran Hospital in Oshikoto Region.

**Methods:**

A qualitative, exploratory, descriptive and contextual design was employed. The accessible population in this study comprised 18 participants who were selected for the study using a purposive sampling technique.

**Results:**

Participants reported numerous barriers to visiting MWHs in Namibia, including an inadequate number of rooms, theft, food scarcity and the effects of poverty on the living conditions of the MWH users. Enablers visiting MWHs included the safe delivery of babies by skilled staff, reduced transport costs, access to timely management of labour complications and affordable accommodation.

**Conclusion:**

The study revealed that a number of barriers must be overcome before the desired number of women take advantage of MWHs. Multiple factors act as constraints to their use, including inadequate number of rooms, theft, food scarcity and the long distance between patients’ homes and MWH services.

**Contribution:**

The study’s findings can be used to develop targeted interventions and strategies that can be used by MWH providers to address the identified barriers.

## Introduction

The global number of pregnancy-related deaths in 2013 was estimated to be 287 000, many of which were preventable.^1^ Consequently, in an attempt to promote maternal and neonatal health, the World Health Organization (WHO) recommended the establishment of maternity waiting homes (MWHs) close to healthcare facilities to ensure safer parturition and/or puerperium periods.^2^ The establishment of MWHs enables easy and timely access to basic maternal healthcare services for childbirth care and the management of obstetric complications at the nearby medical health facility.^3^ From the beginning of the 20th century, MWHs have been built in more than 18 countries around the world, including the United States, Canada, Cuba, India, Nigeria, South Africa, Uganda, Ethiopia and Malawi.^3,4,5^ Despite this effort, issues such as costs, household decision making, knowledge of services and community perceptions of quality of care have remained one of the barriers to successful implementation of MWHs in most countries;^3^ hence, the projection that 390 of every 100 000 pregnant women in sub-Saharan Africa will die during childbirth in 2030.^6^

The childbirth period requires pregnant women to have timely access to a healthcare facility; however, most women in developing countries reside far away from such facilities and must navigate transport issues.^7^ This is especially problematic when a mother is in labour or when complications occurr.^1^ Key players in the health sector should thus advocate for and facilitate the exploration and implementation of different maternal risk reduction measures. For those who can access an MWH, a prolonged stay may to some extent deprives them of physical and psychological support, particularly among those experiencing the birth process for the first time. This, in addition to the financial constraints mothers face while staying in an MWH, could be the reason why few pregnant women use them.

Namibia has a two-tier health system: public health under the Ministry of Health and Social Services (MoHSS) and the private health service. The MoHSS’ vision is to be the leading provider of quality healthcare and social services according to international standards. Since Namibia’s independence, the government has adopted primary health care (PHC) as their approach to providing health services and as a key strategy to attain the goal of good health for all citizens. Namibian health system is also being strengthened by implementing the sustainable development goals (SDGs), which provide a road map for human development and, among others, systematically address the social determinants of health. Namibia is one of the least densely populated countries in the world, with a population of approximately 2.8 people per square kilometre, yet despite this and its status as a middle-income country, Namibia has made inadequate progress towards attaining SDGs 4 and 5.^8^ These goals required improved maternal health and a reduction in the maternal mortality ratio (MMR) by 75%, as well as a reduction in the child mortality rate by two-thirds, from the 1990 level by 2015.^9^ According to the *Confidential Enquiry into Maternal Deaths in Namibia, 2018–2019*, a total of 70 maternal mortalities were reported from 01 April 2018 to 31 March 2019, which was a MMR of 92 for every 100 000 live births.^9^ This was an increase of 48% in comparison with the reported MMR of 2017 (62 of 100 000 live births).^10^

In 2013, Namibia had high perinatal mortality rates of 34 per 1000 pregnancies in the Zambezi region and 32 deaths per 1000 pregnancies in the Kunene region among women who were 7 or more months pregnant.^11^ As a result, the MoHSS, in partnership with WHO and the European Union (EU), launched the Programme for Accelerating the Reduction of Maternal and Child Mortality (PARMaCM), which included the construction of MWHs. An MWH in Opuwo was officially inaugurated in February 2018, yet despite some successes, certain barriers to accessing MWHs still exist for pregnant women, such as long travel distances and a lack of transportation.^12^ As Namibia is a sparsely populated country, with huge distances between health facilities, this situation requires dedicated transportation with vehicles in good condition. The public sector has experienced several challenges in this regard, including a shortage of properly equipped ambulances for emergency responses and the transportation of patients. Linked to this, 18.6% of Namibian women delivered their babies outside health facilities in the year 2013 because of barriers such as the cost of public transportation to reach MWHs.^12,13^ Thus, while MWHs are a critical part of the government’s strategy to reduce MMR as they provide a safe space for women to stay before and after childbirth, their low utilisation rate has the potential to further increase the risk of maternal and newborn deaths and complications.^14,15^ Although several studies have been conducted on the factors influencing women’s access to the MWHs, with some focussing on the factors hindering the use of the MWHs, none have focussed on specifically exploring the utilisations of MWHs in Namibia.^15,16^

## Research methods and design

### Study design

The study utilised a qualitative design that was exploratory, descriptive and contextual in nature. According to Maree et al., a qualitative research design is naturalistic as it focusses on natural settings where interactions occur.^17^ It aims at providing insight into, and an understanding of, the problem faced by the researcher while also describing the characteristics of a problem. In this study, the design enabled the researcher to explore how the participants made sense of their surroundings, their experiences and their understanding of the phenomenon under investigation. It was thus considered an appropriate design for the researcher to explore the perspectives of the pregnant women who utilise the MWHs. Descriptive design was used to help the researcher describe the perspectives of pregnant women on the utilisation of the MWHs. This study was contextual in nature because perspectives of pregnant women on the utilisation of the MWHs were described in relation to Onandjokwe Lutheran Hospital setting.

### Study setting

This study was conducted at Onandjokwe Lutheran Hospital, a peri-urban hospital, serving a large catchment area in the Oshikoto region in north-western Namibia. The hospital serves as a referral hospital for the region and delivers about 700 babies monthly and an average of 7668 babies annually. The Onandjokwe MWH is located outside the hospital but is not a part of the hospital; it is, however, built very close to the boundary of the hospital. It allows pregnant women to access quality maternal and newborn care by bringing those living in remote areas closer to health facilities for a safer childbearing process. The Onandjokwe MWH admits expectant mothers both primigravidae and multigravida from nearby regions like Ohangwena and Oshana. On a monthly basis, the MWH admits a total of 220 pregnant women who are in the last trimester of pregnancy – both low and high risks. In addition, the MWH also has an assigned registered midwife on call who oversees administrative functions such as admissions, discharges and the referral of women about to commence labour or those who are unwell.

### Sample and sampling technique

The study participants were pregnant women who had been admitted to the Onandjokwe Lutheran MWH prior to delivery. Purposive sampling was used to select potential participants according to the following inclusion criteria: (1) in the last trimester of their pregnancy awaiting delivery at the MWH; (2) willing to participate and (3) agreed to provide the researcher with written informed consent. The following exclusive criteria were used to exclude participants namely: (1) pregnant women awaiting delivery at the MWH who meet the inclusive criteria but were not available at the time of data collection, (2) pregnancy women awaiting delivery at the MWH who meet the inclusive criteria but did not consented to take part in the study and (3) pregnancy women awaiting delivery at the MWH who are not in their last trimester of their pregnancy. Data saturation was reached at the 18th participant. At this point, the researchers ceased the data collection process as no new data on the phenomenon under investigation would have surfaced.

### Data collection methods

The researcher approached the potential participants in person between October 2022 and December 2022. Data were collected through individual, semi-structured interviews in accordance with an interview guide that was developed based on three aspects: the research objectives, the literature review and the central research question. In addition, three pilot interviews were initially conducted with similar participants in order to check for possible errors and capacity to gather the relevant data. Thereafter, no amendments were made to the interview guide, and the data generated from the pilot interview were not included in the main study because the pilot interview was conducted merely to test the interview guide for possible errors that may warrant a need for adjustment of the interview guide before the actual data collection. Prior to participating in the study, the researcher explained the aim and significance of the research to the participants and their right to withdraw at any time without any coercion. The willing participants signed an informed consent form before participating in the study. Furthermore, the researcher also sought permission from the participants to record the interviews. Given that the majority of participants were educated, a semi-structured interview schedule was conducted in English and lasted 40 min – 45 min each. Probing questions were posed to encourage the participants to expand on their responses. The central questions asked were:

- What is your opinion of the utilisation of the MWH by pregnant women at Onandjokwe Lutheran Hospital in Oshikoto region, Namibia?
- What made you want to stay here?
- What would make you not want to stay here?
- What suggestions do you have to improve the utilisation of the MWH?

### Data analysis

The audio-recorded interviews were transcribed verbatim for concurrent data collection and analysis with manual coding, which involves reading over each comment and manually assigning labels. Basic demographic details of participants were collected. The researcher used an inductive approach to analyse the data, using the thematic analysis technique. The researcher used the reflexive thematic analysis approach, which focusses on people’s experiences, views, perceptions and representations of a particular phenomenon.^18^ Braun and Clarke’s six phases of thematic analysis were employed: Step (1) Familiarisation (getting to know the data); Step (2) Coding; Step (3) Generating themes; Step (4) Reviewing the themes; Step (5) Defining and naming themes and Step (6) Writing up the analysis and generating a report.^19^ The researcher documented his own views and previous knowledge of the phenomenon before collecting data so that he was able to engage in the self-reflective process of ‘bracketing’, whereby the researcher is expected to distinguish and set aside (but not abandon) his/her *a priori* knowledge and assumptions, with the goal of being open minded when listening to the participants.^20^ The author read the field notes in conjunction with the transcribed data as the data analysis process unfolded. A coding tree was designed by the first author, which highlighted the themes and described how each sub-theme developed. The coding tree also clarified which codes formed which themes. This was used to understand how the themes were created and showed that the themes came from the data as opposed to being selected beforehand. The author then resolved which themes and sub-themes would be used in their reporting. An independent coder who was not one of the co-authors undertook an inquiry audit, and a consensus was reached on the themes and sub-themes. This involved an analysis of the coding tree, the transcribed data and the field notes to establish that each of the themes was extracted from the collected data. The independent coder had comprehensive experience in qualitative research and was also a nurse educator with a Doctorate Degree in Nursing Science.

### Trustworthiness

The trustworthiness of the whole study was ensured by using Lincoln and Guba’s model, which ensures the credibility, dependability, confirmability and transferability of a study.^21,22^ Credibility was realised by holding pilot interviews, establishing data saturation, prolonging participant engagement as the researcher remained in the field for 8 weeks, actively participating, observing and interacting with participants, recording the interviews, member checking (by replaying the recordings to the participants) and validating the transcripts with the research supervisors. In this study, interviews were performed by the researcherwho is affiliated to the Onandjokwe Lutheran Medical Hospital. Consequently, participants may have answered differently. In respect to this relationship, the main dilemma stemmed from the fact that the researcher was a District Primary Health Care – Supervisor interviewing pregnant women awaiting delivery at the MWH. The researcher adopted neutrality of the relationship between the participants and the researcher. However, because of power-relation between researcher and pregnant woman awaiting delivery, a hierarchical barrier framing the exchange of information will always remain. To ensure that pregnant woman awaiting delivery provided honest feedback, the researcher developed a trusting relationship that could enable the pregnant woman awaiting delivery to freely express their perspectives of pregnant women on the utilisation of the MWHs. To achieve this, the researcher remained open to all possible interpretations of the events and encouraged pregnant women awaiting delivery to provide honest feedback. The researcher also openly adopted a non-judgemental stance and as a result pregnant women awaiting delivery freely revealed their responses. This unbalanced relationship is systematically considered during the research, particularly in the interpretation of data phase. Whenever pregnant women awaiting delivery expressed their perspectives on the utilisation of the MWHs, the researcher wondered if their statements were geared to satisfy the researcher. Past experiences with interviews have shown us that because of the power relation between patients and nurses especially who are affiliated to the ministry or government agency such as the hospital, one of the two tends to provide widely positive feedback. Therefore, it has been argued that whatever the respondents say should be treated as valid.^23^ However, this should be subject to caution in asymmetrical relationships such as that of a nurse and pregnancy awaiting women.

Transferability was achieved by incorporating rich descriptions of the experiences of the expectant mothers, as well as their contexts, so that their meaning was evident to outsiders.^24^ Dependability was ascertained via a peer debriefing with researchers not involved in the study, as well as extended engagement with the interviewees and member checking. An inquiry audit using an external reviewer ensured conformability. Finally, reflexivity was established by the researchers remaining aware of their roles in the study and their ongoing reflection on their personal behaviours and how these might affect the research.^22^ This reflection was made possible through the use of research diaries, which the authors incorporated into their field notes.

### Ethical considerations

The researcher obtained permission to conduct the study from the University of Namibia (ethical clearance number: SoNREC 22/2022) as well as the Ministry of Health and Social Services (Ref: 17/3/3/AVN) before the study was conducted. In addition, participation in the study was entirely voluntary, with each participant consenting in writing before beginning. No incentives were offered during the process of attaining participants and inclusion criteria were enforced to mitigate any potential bias. Anonymity was established by giving each participant a number, which replaced their names. Finally, confidentiality was ensured by keeping all of the interview recordings on password-protected devices, which only the researchers had access to. There were no anticipated risks for participating in this study as the study did not involve any treatment or interventions. There was no direct benefit for participating in this study, but it is expected that the results of this study can help to explore and describe the perspectives of pregnant women on the utilisation of the MWHs at Onandjokwe Lutheran Hospital in Oshikoto region, Namibia, and formulate targeted interventions and strategies that can be used by MWH providers to address the identified barriers.

## Results

### Demographic characteristics of the participants

There were 18 participants in total, aged between 15 years and 45 years. The majority were between 15 years and 25 years. Of the 18 participants, 16 were single and the remaining 2 were married. Most (*n* = 14) of the study participants had a secondary education, while two had a tertiary education and two had no education (Table 1).

**TABLE 1:** Demographic characteristics of the participants.

Participant #.	Age	Marital status	Educational level	Employment status
P1	26	Single	Grade 10	Unemployed
P2	23	Single	Grade 10	Unemployed
P3	24	Single	Grade 10	Unemployed
P4	20	Single	Grade 10	Unemployed
P5	24	Single	Tertiary education	Domestic worker
P6	28	Single	Grade 8	Unemployed
P7	18	Single	No formal schooling	Unemployed
P8	33	Single	Tertiary education	Self-employed
P9	37	Single	Grade 10	Unemployed
P10	22	Single	Grade 9	Unemployed
P11	37	Single	Grade 10	Unemployed
P12	39	Single	Grade 9	Unemployed
P13	43	Married	Grade 12	Unemployed
P14	23	Single	Grade 9	Unemployed
P15	23	Single	Grade 11	Unemployed
P16	33	Married	Grade 8	Unemployed
P17	26	Single	No formal schooling	Unemployed
P18	27	Single	Grade 10	Unemployed

The reflexive thematic analysis resulted in 3 main themes and 13 sub-themes being extracted, which are presented in Table 2.

**TABLE 2:** Summary of findings: Themes and sub-themes.

Themes	Sub-themes
1. Enablers for the utilisation of MWHs	1.1 Safe delivery by skilled staff at the health facility 1.2 Reduced transport costs 1.3 Access to timely management of labour complications 1.4 Affordable accommodation
2. Challenges to the utilisation of MWHs	2.1 Inadequate number of rooms 2.2 Theft 2.3 Scarcity of food 2.4 Poverty and living conditions of users
3. Recommendations to increase the use of MWHs	3.1 Increase in number of rooms 3.2 Strengthen safety and security measures 3.3 Provision of food grants from government 3.4 Improvement in maintenance of infrastructure 3.5 Provision of hot water

MWH, maternity waiting home.

### Theme 1: Enablers for the utilisation of maternity waiting homes

Theme 1 relates to the identified factors that play a role in facilitating the utilisation of the MWH. Participants mentioned the perceived benefits as being the high quality of care at the health facility and the benefits of staying at the MWH. The sub-themes under theme 1 are safe delivery by skilled staff at the health facility, reduced burden on transport cost, access to timely management of labour complications and affordable accommodation.

#### Sub-theme 1.1: Safe delivery by skilled staff at the health facility

The participants noted that it is safer to stay at the MWH because it is close to the hospital; if they experience any complications, doctors and nurses are readily available. The participants also highlighted the fact that a big hospital like Onandjokwe Intermediate Hospital has better medical equipment than district hospitals and clinics:

‘I feel lucky and safe because I am closer to the health care services [*nurses and doctors*] … I could say the maternity waiting house helps prevent home delivery.’ (P1, 26 years old, Single)

‘We also benefited as we have access to maternal health services hospital … like Onandjokwe are big with good medical equipment.’ (P18, 27 years old, Single)

‘It saved me from transportations delays complication to be attended in the hospital.’ (P3, 24 years old, Single)

#### Sub-theme 1.2: Reduced transport costs

The participants stated that they are from rural areas with poor road infrastructure, thus staying at the MWH reduces the burden of travel costs during an emergency:

‘The maternity waiting house helps us to be closer to the hospital and save … us from unnecessary travelling in order to see the doctor and thus saving us some money.’ (P1, 26 years old, Single)

‘I am grateful, because not all hospital has MWH like Onandjokwe and we do not have to suffer finding transport money to go to hospital when labour starts.’ (P 11, 37 years old, Single)

‘I get to settle here while waiting for the arrival of our babies and not having to walk long distances to reach the hospital when labour start.’ (P8, 33 years old, Single)

#### Sub-theme 1.3: Timely management of complications

Participants expressed the advantages of living in the MWH while pregnant. They stated that it maximises the chances of survival of both mother and baby if maternal complications arise:

‘I am grateful, because here I am close to the hospital. Access to doctor and nurses all the time I need immediately medical assistance.’ (P17, 26 years old, Single)

‘I feel good to have this opportunity. I have seen in many instances other pregnant women give birth at home because of distances to reach hospital and ended up in complications after delivery.’ (P16, 33 years old, Married)

‘I could say the maternity waiting house helps prevent home delivery, which could be a complication for those that live deep in villages’ (P1, 26 years old, Single

#### Sub-theme 1.4: Affordable accommodation

The participants expressed their satisfaction with the fees they must pay during their stay at the MWH. They indicated that if they cannot afford to pay the prescribed fees on a daily basis, the management allows them to pay in kind via services like cleaning:

‘We only pay two Namibian (N$2.00).’ (P5, 25 years old, Single)

‘We pay two Namibian dollars upon arrival and if you do not have money to pay you then have to clean as payment.’ (P10, 22 years old, Single)

‘We pay two Namibian dollar or weed if we don’t have money.’ (P5, 24 years old, Single)

### Theme 2: Challenges to utilisation of maternity waiting homes

The participants mentioned several barriers that affect their utilisation of the MWH. The sub-themes under this theme are insufficient rooms for accommodation, increased cases of theft at the MWH, scarcity of food and the effects of poverty on the living conditions of the users.

#### Sub-theme 2.1: Inadequate number of rooms

Some participants mentioned that insufficient rooms are available to accommodate all the pregnant women, which is a major barrier to the utilisation of the MWH. One participant commented on the fact that some women sleep outside because of a lack of space, while another participant spoke about how some women sleep on the floor:

‘All rooms are fully occupied; some people are sleeping outside because of space.’ (P1, 26 years old, Single)

‘We are eight in the room but some are six or seven and since most of the time the rooms are fully occupied some pregnant women are forced to sleep outside and on the floor.’ (P5, 24 years old, Single)

‘The waiting house is small and cannot accommodate all of the pregnant women [*some sleep in their own tents outside the waiting house*].’ (P2, 23 years old, Single)

#### Sub-theme 2.2: Theft

Participants also expressed concerns regarding theft by others in the MWH:

‘Other pregnant women steal other women’s property and food.’ (P1, 26 years old, Single)

‘I observed that kitchens are of poor structure, without stoves and has no lockers for everyone to keep their food to prevent theft.’ (P9, 22 years old, Single)

‘I also experienced mothers stealing other’s food as well as properties, such as phones’ (P11, 37 years, Single)

#### Sub-theme 2.3: Scarcity of food

The participants expressed a concern regarding the lack of food during their stay at the MWH, which sometimes contributes to food theft:

‘With regards to food, we come with our own food and when they get finish we send from home.’ (P4, 20 years old, Single)

‘I do not have enough food and sometimes I stay without food or I beg from my colleagues.’ (P3, 24 years old, Single).

‘We then cook and eat as a group that way the food lasts compared to when you are alone and if the food gets finished we sleep on empty stomachs’ (P9, 37 years old, Single)

#### Sub-theme 2.4: Poverty and living conditions of users

As the majority of the participants were unemployed and many had travelled long distances to get to the home, staying at the MWH had financial implications. Some participants highlighted the importance of forming small communities within the waiting home in order to pool resources, while some expressed the difficult circumstances under which the women must live:

‘Some of us we grouped ourselves into three or four and put our food together. We then cook and, in the group, that way the food lasts compared to when you are alone and if the food gets finished we sleep on empty stomachs. At times we get soft porridge from the hospital kitchen.’ (P8, 33 years old, Single)

‘It is difficult to survive here, because we have to come with our own food but they get stolen by those that do not have.’ (P17, 26 years old, Single)

‘We use open fire to cook, but sometimes we do not have firewood which we buy and it is expensive.’ (P14, 23 years old, Single)

### Theme 3: Recommendations to increase the use of maternity waiting homes

Participants suggested some improvements that would make the MWH a more conducive environment for the pregnant women living there, which led to the emergence of the following sub-themes: increase the number and/or size of rooms, strengthen security measures, provide food, renovate kitchen stoves and cupboards and provide hot water.

#### Sub-theme 3.1: Increase in the number of rooms

Participants suggested that the government must consider expanding the MWH:

‘The Ministry should enlarge the house to reduce the number of pregnant women sleeping outside.’ (P7, 18 years old, Single)

‘I would say that it will be better if the hospital plan for the MWH enlargement to be able to accommodate more pregnant women.’ (P8, 33 years old, Single)

‘Here sometimes rooms are fully occupied some people I even sleeping outside, I suggest that people should start coming with sleeping tents for sleeping.’ (P2, 23 years old, Single)

#### Sub-theme 3.2: Strengthening safety and security measures at the maternity waiting home

The participants expressed their concern regarding their compromised safety at the MWH. They recommended that the hospital management assign a designated security officer to the waiting home as there is currently no security guard stationed there. In addition, the participants recommended installing lockable cupboards:

‘I suggest that the hospital should allocate a security personnel to guard the behaviours of the pregnant women in the MWH because some pregnant are stealing other occupants’ items.’ (P2, 23 years old, Single)

‘I’m suggesting that the hospital management must renovate the cupboard and put a provision of padlocks and cupboard locks to reduce theft.’ (P6, 28 years old, Single)

‘I recommend that the hospital must put up security fences to keep away animals eating away our foods at night.’ (P16, 33 years old, Married)

#### Sub-theme 3.3: Provision of food grants from the government

The participants suggested that mothers who are admitted to the MWH be provided with food by the government, as most of them travel long distances. When their food is finished it is difficult for them to obtain more. The provision of food by the state would also help the victims of food theft:

‘I feel the government needs to assist us with food because some pregnant women come from far and it is not easy to get food from home.’ (P2, 23 years old, Single)

‘The government should at least help provide food sometimes for the pregnant because sometimes we sleep with empty stomachs.’ (P4, 20 years old, Single)

#### Sub-theme 3.4: Improvement in infrastructure maintenance

The participants suggested that the kitchen needs renovation as it is dilapidated and the stoves do not function. Further suggestions for improvement included the maintenance and repair of the lockable cupboards, as well as a padlock for the main entrance to the kitchen:

‘The Ministry should enlarge the house to reduce the number of pregnant women sleeping outside and the hospital management should renovate the cupboard and put a provision of padlocks.’ (P6, 28 years old, Single)

‘I want the government to renovate the kitchen cupboard, expand showers with provision of hot water; the government should expand the maternity waiting house.’ (P11, 37 years old, Single)

‘I would say that it will be better if the hospital plan for the maternity waiting home enlargement to be able to accommodate more pregnant women.’ (P7, 18 years old, Single)

#### Sub-theme 3.5: Provision of hot water

Participants expressed challenges regarding the lack of hot water, especially during winter. They mentioned that:

‘We need provision of hot water, especially during winter; cold water makes us suffer from hypothermia and reduces foetal movements.’ (P10, 22 years old, Single)

‘I want the government to renovate the kitchen cupboard, expand showers with provision of hot water.’ (P15, 23 years old, Single)

‘We need provision of hot water, especially during winter.’ (P17, 26 years old, Single)

## Discussion

This study aimed to explore and describe the perspectives of pregnant women regarding the utilisation of the MWH near Onandjokwe Lutheran Hospital in the Oshikoto Region. According to the findings, the use of MWHs by pregnant mothers come with a wide range of benefits, from affordable accommodation and timely management of complications, to reduced transport costs and safe delivery by skilled staff. In addition, MWHs are the best places for pregnant women to wait for their delivery because of their closeness to a maternity ward, which provides easier and more timely access to skilled midwives and medical officers.^5^ As a result, the use of MWHs can be strengthened by providing upfront aid for transportation costs and community involvement to identify cultural barriers.^3,24,25^ The use of community involvement to strengthen MWHs as aid for transportation costs was interesting because this aspect did not surface in the current study. Women’s use of MWHs may have increased because of healthcare workers’ advice during antenatal care visits.^26^ Penn-Kekana et al.^3^ indicated that facilitators include lowering or abolishing costs related to utilising an MWH, community participation in the design and maintenance of the MWHs, community events to promote awareness and acceptance among relatives and community members, as well as incorporating necessary cultural practices into rendering maternal and new-born care at the MWHs and healthcare facilities.

Participants were also appreciative of living in MWHs where they had access to higher levels of obstetric care without incurring the costs of private transport during labour. Additionally, staying in MWHs provides a safe and comfortable space where they can stay in the last weeks of pregnancy and allows more women to deliver their babies at nearby health centres that are equipped to handle potential labour complications and provide quality care to newborns.^27,28^ According to the participants, a timely intervention during a possible pregnancy complication has the potential to maximise the chances of survival of both mother and baby. In more remote areas, it is thus important to establish more MWHs in order to create a reliable referral system and to provide basic emergency obstetric and newborn care services to communities.^15^ However, it also emerged that the quality of MWHs and their services need to be strengthened to attract more women and improve maternal-neonatal safety. In addition, while our study did not reveal facilitators for community awareness, Penn-Kekana et al. reported that decentralising MWH services also requires raising community awareness to create a positive attitude and acceptance, which will enhance the utility of these facilities by the targeted population.^3^

The use of MWHs was also associated with several shortcomings, which are potential barriers to the future use of MWHs. These drawbacks included theft, scarcity of food and the effects of poverty on living conditions. In addition, the long distances between the women’s homes and the MWH, transport costs, the cost of living in MWHs, inadequate bathrooms and kitchens and insufficient room for family and companions could be additional reasons for why women may turn up late to the MWH or opt to deliver home.^3,29,30^ Participants raised a concern about limited space, which leads to some having to share sleeping rooms while others having to sleep outside. Some mothers may also opt not to stay in an MWH because of an unwillingness to be away from their family or employment for an extended period.^30^ Geleto et al. also indicated that a potential barrier towards the use of MWHs may be insufficient funding as a result of unemployment, that is, women are unable to sustain their needs while staying in an MWH.^29,31^ Additional barriers to using MWHs include a lack of cooking utensils, inconsistent supplies of electricity and water, inadequate sanitation facilities and family decision-making process of women and their families for using an MWH involves balancing out the gains and losses.^5,32,33,34^

As there is a limited number of MWHs in the country, the government ought to consider establishing more while strengthening the existing safety and security measures. In addition, there is a need to improve the livelihoods and welfare of potential occupants to attract more pregnant women. Incorporating a culturally sensitive and supportive maternal healthcare services and community engagement approach will also have a significant impact on the use of MWHs, thereby promoting maternal health.^35^ The lack of awareness of MWHs can be addressed by providing health education and empowerment among pregnant women.^36^

Similar to the participants expressing concerns regarding the theft of property and food because of a lack of lockable lockers, Chibuye et al.^5^ highlighted the need to strengthen privacy and security measures in order for MWHs to reach their intended potential. In addition, some participants had to face financial constraints during their stay in the MWH as they were not employed and had to travel long distances to the waiting home. According to Bergen et al., by providing food, transport and grants more pregnant women may be motivated to stay at an MWH.^33^

### Strengths and limitations

This study provides insight into the challenges faced by pregnant women regarding their use of MWHs. The explorative design enabled the participants to freely narrate and interpret their experiences, as well as make suggestions for improvements. As only women attending an MWH were interviewed, the researcher did not gather data from those who had not utilised such facilities. Regarding the interview guide, the limitation stems from the fact that it would have been interesting to ask participants if they would use such a facility again in the future or maybe there were some who were back after a previous delivery and why did they come back. Finally, although the research data cannot be generalised to other healthcare facilities in the country, a significant lesson can be learnt in terms of provision of maternity care.

### Recommendations

Based on the findings of this study, the following recommendations are made to support the MWHs:

- There should be facility messages to community members that are clear and consistent in bringing the community together to support the MWHs.
- There is a need to strengthen programmes coordinated by chiefs and headmen of the villages for supporting birth planning, prevention of early marriage and other women and girls’ health initiatives with a view to improving planning of birth.
- Build upon the continued work of community promotors such as health extension workers and other community health workers for referrals, promoting skilled delivery and empowering women and families to improve financial planning for birth.
- Continue community sensitisation efforts with consistent messaging through other community development funds and other partners with a view to improve community’s ability to build and maintain physical structure.

## Conclusion

The findings of this study highlight the perceptions of the participants regarding the quality of care at the maternal waiting home at Onandjokwe Lutheran Hospital in Namibia. Perceived benefits include safe deliveries by skilled staff, reduced transport costs, access to timely management of labour complications and affordable accommodation. On the other hand, they described several barriers that affect their utilisation of the MWH, including an inadequate number of rooms, theft, food scarcity and the effects of poverty on living conditions. These findings can be used to develop ongoing strategies and targeted interventions that are geared towards increasing the utilisation of the MWHs. As a result, this study recommends adding rooms, improving the sanitation facilities, strengthening security measures and supplying consistent electricity to enhance the utility of MWHs. In addition, it is important that the ministry provides food or grants to the occupants of MWHs, as well as cooking utensils.
